# Psychiatric comorbidity patterns across disability-based fibromyalgia phenotypes

**DOI:** 10.1017/S1092852926100893

**Published:** 2026-03-24

**Authors:** Kevin V. Hackshaw, Michelle M. Osuna-Diaz, Katherine R. Sebastian, Shreya Madhav Nuguri, Silvia deLamo Castellvi, Lianbo Yu, M. Monica Giusti, Zhanna Mikulik, Luis Rodriguez-Saona

**Affiliations:** 1 Medicine, The University of Texas at Austin Dell Medical School, USA; 2 Food Sciences and Technology, The Ohio State University, USA; 3 Campus Sescelades, Departament d’Enginyeria Química, Universitat Rovira i Virgili, Av. Països Catalans 26, 43007 Tarragona, Spain; 4 Bioinformatics, The Ohio State University Wexner Medical Center, USA; 5 Medicine, The Ohio State University Wexner Medical Center, USA

**Keywords:** Fibromyalgia, psychiatric comorbidity, affective symptoms, disability, dimensional psychopathology

## Abstract

**Objective:**

Fibromyalgia (FM) is a heterogeneous chronic pain condition frequently accompanied by psychiatric symptoms. Although affective symptoms are highly prevalent, they are often treated as secondary correlates of pain or disability. This study examined whether psychiatric symptom profiles parallel disability severity or represent partially independent dimensions across disability-based FM phenotypes.

**Methods:**

We analyzed a harmonized multisite cohort of adults with FM recruited from two academic medical centers. Disability-based phenotypes were defined using a validated percentile-based classification dichotomizing participants into low-impact and high-impact FM. Psychiatric domains were derived from standardized measures of depressive and anxiety-related symptoms. Multivariate clustering was used to identify affective profiles, and their distribution across disability phenotypes was examined. Dimensional analyses assessed the relationship between affective burden and functional impairment.

**Results:**

Among participants with complete psychiatric data (*n* = 613), four reproducible affective profiles were identified: minimal affective symptoms, mild affective symptoms, moderate mixed affective symptoms, and severe mixed affective symptoms. Although profiles characterized by greater affective burden were enriched among individuals with high-impact FM, all affective profiles were represented across both disability groups. Notably, a substantial proportion of individuals with high-impact FM exhibited minimal or mild affective symptoms. Dimensional analyses supported partial orthogonality between affective burden and disability severity.

**Conclusions:**

Psychiatric comorbidity in FM does not simply reflect pain severity or functional impairment. Instead, affective symptoms form partially independent dimensions that cut across disability-based phenotypes. These findings support a multidimensional neuropsychiatric framework for FM with implications for stratified assessment and personalized intervention.

## Highlights

- Fibromyalgia psychiatric comorbidity forms reproducible affective profiles rather than scaling uniformly with disability.
- Four-dimensional affective profiles were identified, ranging from minimal to severe mixed affective symptoms.
- Psychiatric profiles cut across disability strata, demonstrating partial independence from functional impairment.
- These findings support a multidimensional neuropsychiatric model of fibromyalgia.

## Introduction

Fibromyalgia (FM) is a chronic pain condition characterized by widespread pain, fatigue, sleep disturbance, cognitive complaints, and impaired physical and social functioning.^1^ ^,^^2^ ^,^^3^ ^,^^4^ ^,^^5^ In addition to somatic symptoms, psychiatric comorbidities—particularly depressive and anxiety-related symptoms—are highly prevalent and contribute substantially to disease burden and reduced quality of life.^6^ ^,^^7^ ^,^^8^ Despite this, psychiatric symptoms in FM are often conceptualized as secondary consequences of pain severity or disability rather than as defining features of illness heterogeneity. FM is highly comorbid with anxiety and depressive disorders, with prevalence estimates ranging from 40 to 70%, substantially exceeding rates observed in the general population. These associations are bidirectional and reflect overlapping neurobiological, psychosocial, and behavioral mechanisms, including altered stress responsivity, central sensitization, affective dysregulation, and maladaptive coping processes. Importantly, psychiatric symptoms in FM are not fully explained by pain severity or functional limitation, suggesting partially independent affective dimensions that contribute to overall disease burden and disability. Large observational studies and meta-analyses have demonstrated that affective symptoms independently predict quality of life, healthcare utilization, and treatment response in FM.^6^ ^,^^7^ ^,^^8^

Recent data-driven studies have demonstrated that FM symptoms organize into reproducible phenotypes primarily distinguished by overall symptom burden and functional impairment.^9^ ^,^^10^ ^,^^11^ These disability-based phenotypes have shown stability across cohorts, suggesting that FM heterogeneity reflects graded differences in global disease impact rather than idiosyncratic symptom constellations.^12^ Within this framework, psychiatric symptoms are frequently included as covariates or mediators in analyses focused on pain mechanisms or cross-condition comparisons.^13^ ^,^^14^

However, contemporary neuropsychiatric models of chronic pain emphasize interactions between nociplastic pain mechanisms, affective regulation, and salience processing, implicating partially overlapping but distinct neural systems.^15^ ^,^^16^ ^,^^17^ From this perspective, affective symptom burden may vary independently of pain intensity or functional impairment, giving rise to distinct psychiatric profiles within similar disability strata.^18^

Clarifying whether psychiatric comorbidity in FM parallels disability severity or represents a partially independent axis has both mechanistic and clinical relevance. If affective symptoms simply scale with disability, they may be adequately captured by global severity indices. Conversely, if affective profiles cut across disability phenotypes, they may warrant independent assessment and targeted intervention.

The present study reframes psychiatric symptoms in FM as primary features of illness heterogeneity rather than secondary correlates. Using a large, harmonized multisite dataset, we examined psychiatric comorbidity patterns across disability-based FM phenotypes. We hypothesized that affective symptom profiles would demonstrate partial independence from disability severity, supporting a multidimensional neuropsychiatric model of FM. The primary objective of this study was to determine whether psychiatric symptom patterns parallel disability severity or represent partially independent dimensions across disability-based FM phenotypes. We hypothesized that (1) reproducible affective profiles would emerge, (2) these profiles would not map uniformly onto disability strata, and (3) dimensional analyses would demonstrate partial orthogonality between affective burden and disability severity.

## Methods

### Participants and dataset

This cross-sectional analysis used a harmonized dataset of adults with FM recruited from two geographically distinct academic medical centers. All participants met established clinical criteria for FM and completed standardized self-report assessments of pain, functional impairment, central sensitization, and psychiatric symptoms. Participants without available disability classification were excluded from phenotype-based analyses. Adults were between the ages of 18 and 80 years meeting the 2016 American College of Rheumatology FM criteria and were recruited from The University of Texas at Austin and The Ohio State University.^12^ ^,^^19^ Recruitment occurred from September 2020 to October 2025 as part of an ongoing biomarker and phenotyping study. All participants provided written informed consent, and institutional review board approval was obtained at both sites (supervising IRB: UT Austin IRB #2020030008). Although recruitment remains ongoing, the analytic dataset for the present study was frozen on October 15, 2025, and includes all participants enrolled through that date with completed disability classification.

### Disability-based fibromyalgia phenotypes

Disability-based phenotypes were defined using a previously validated percentile-based disability classification, dichotomizing participants into low-impact and high-impact FM groups.^20^ ^,^^21^ This approach provides a clinically interpretable stratification of functional impairment.

### Psychiatric measures

Depressive symptom burden was assessed using the Beck Depression Inventory, a widely validated self-report instrument that captures cognitive, affective, and somatic dimensions of depressive symptoms.^22^ ^,^^23^ Affective distress and emotional functioning were assessed using the Emotional Well-Being subscale of the SF-36, which reflects global psychological distress and perceived emotional health.^24^ Although the SF-36 is not a diagnostic psychiatric instrument, its emotional well-being domain has demonstrated sensitivity to affective burden in chronic illness populations and is frequently used in FM research.

These instruments were selected to capture dimensional affective symptom severity rather than categorical psychiatric diagnoses, aligning with contemporary dimensional models of psychopathology and allowing characterization of affective profiles within a heterogeneous clinical population. These measures were standardized prior to multivariate analyses.

Psychiatric Profile Derivation Cluster solutions ranging from two to six clusters were evaluated. The four-cluster solution was supported by an average silhouette width of 0.41, elbow-method inspection of within-cluster sum of squares, bootstrap stability across 1000 resamples, and clinical interpretability.

A four-profile solution was selected based on stability across resampling procedures, clinical interpretability of the resulting symptom patterns, and consistency with prior clustering approaches in FM and chronic pain populations. Solutions with fewer profiles failed to capture meaningful affective heterogeneity, whereas higher-order solutions resulted in fragmentation without clear clinical relevance. The retained profiles demonstrated coherent gradations of affective burden and remained stable across analytic specifications.

Multivariate clustering (k-means) was applied to standardized depressive and anxiety-related variables among participants with complete psychiatric data. This approach yielded four affective profiles: minimal affective symptoms, mild affective symptoms, moderate mixed affective symptoms, and severe mixed affective symptoms.

### Statistical analysis

The distribution of psychiatric profiles across disability phenotypes was examined using contingency tables and chi-square testing. Principal component analysis was used to examine the dimensional relationship between affective burden and functional disability. Analyses were conducted using R, with statistical significance set at α = 0.05.^25^ The first principal component explained 48.3% of total variance and loaded primarily on disability and pain variables, while the second principal component explained 27.1% of variance and loaded primarily on affective variables. AI-assisted tools were used for initial statistical script drafting; however, all analytic procedures were independently reviewed and verified by a biostatistician to ensure reproducibility and methodological accuracy.

## Results

Results are presented to first describe cohort characteristics, followed by the derivation of affective profiles and their distribution across disability-based FM phenotypes.

### Sample characteristics

The analysis cohort included 695 participants with available disability classification (520 low-impact FM; 175 high-impact FM). Demographic and clinical characteristics across disability phenotypes are summarized in Table 1.

**Table 1.** Demographic and Clinical Characteristics Across Disability-Based Fibromyalgia Phenotypes

Variable	Low-Impact Fibromyalgia	High-Impact Fibromyalgia
Age, years (mean ± SD)	45.2 ± 15.6	45.1 ± 12.1
Female sex, *n* (%)	446 (86.3%)	169 (96.6%)
Body mass index (mean ± SD)	30.6 ± 8.3	34.5 ± 10.1
SF–36 physical component summary (mean ± SD)	32.4 ± 9.9	23.0 ± 5.4
Pain score (mean ± SD)	42.1 ± 22.2	15.8 ± 13.7
Central sensitization inventory score (mean ± SD)	50.7 ± 17.8	72.2 ± 12.0
Beck depression inventory score (mean ± SD)	14.4 ± 9.2	27.2 ± 10.2

*Note*: Demographic and clinical characteristics of participants with low-impact and high-impact fibromyalgia. Continuous variables are presented as mean (standard deviation), and categorical variables are presented as number (percentage). Disability-based phenotypes were defined using a validated percentile-based classification. Group comparisons were performed using independent-samples t tests or χ^2^ tests, as appropriate.

### Psychiatric comorbidity profiles

Among participants with complete psychiatric data (*n* = 613), clustering identified four reproducible affective profiles: minimal affective symptoms, mild affective symptoms, moderate mixed affective symptoms, and severe mixed affective symptoms.

### Distribution across disability phenotypes

The distribution of affective profiles across disability-based FM phenotypes is shown in Table 2. Although profiles characterized by moderate and severe affective burden were enriched among individuals with high-impact FM, all affective profiles were represented across both disability groups. Notably, 38 participants with low-impact FM exhibited severe mixed affective symptoms, while 38 participants with high-impact FM exhibited minimal or mild affective symptoms.

**Table 2.** Distribution of Psychiatric Affective Profiles Across Disability-Based Fibromyalgia Phenotypes

Psychiatric Affective Profile	Low-Impact Fibromyalgia (n)	High-Impact Fibromyalgia (n)
Minimal affective symptoms	146	10
Mild affective symptoms	159	28
Moderate mixed affective symptoms	107	70
Severe mixed affective symptoms	38	55
Missing psychiatric data	70	12

*Note*: Distribution of data-driven psychiatric affective profiles across low-impact and high-impact fibromyalgia phenotypes among participants with complete psychiatric data (*n* = 613). Psychiatric profiles were derived using multivariate clustering of standardized depressive and anxiety-related symptom measures and categorized as minimal affective symptoms, mild affective symptoms, moderate mixed affective symptoms, and severe mixed affective symptoms.

### Dimensional relationship between affective burden and disability

**Figure 1.**
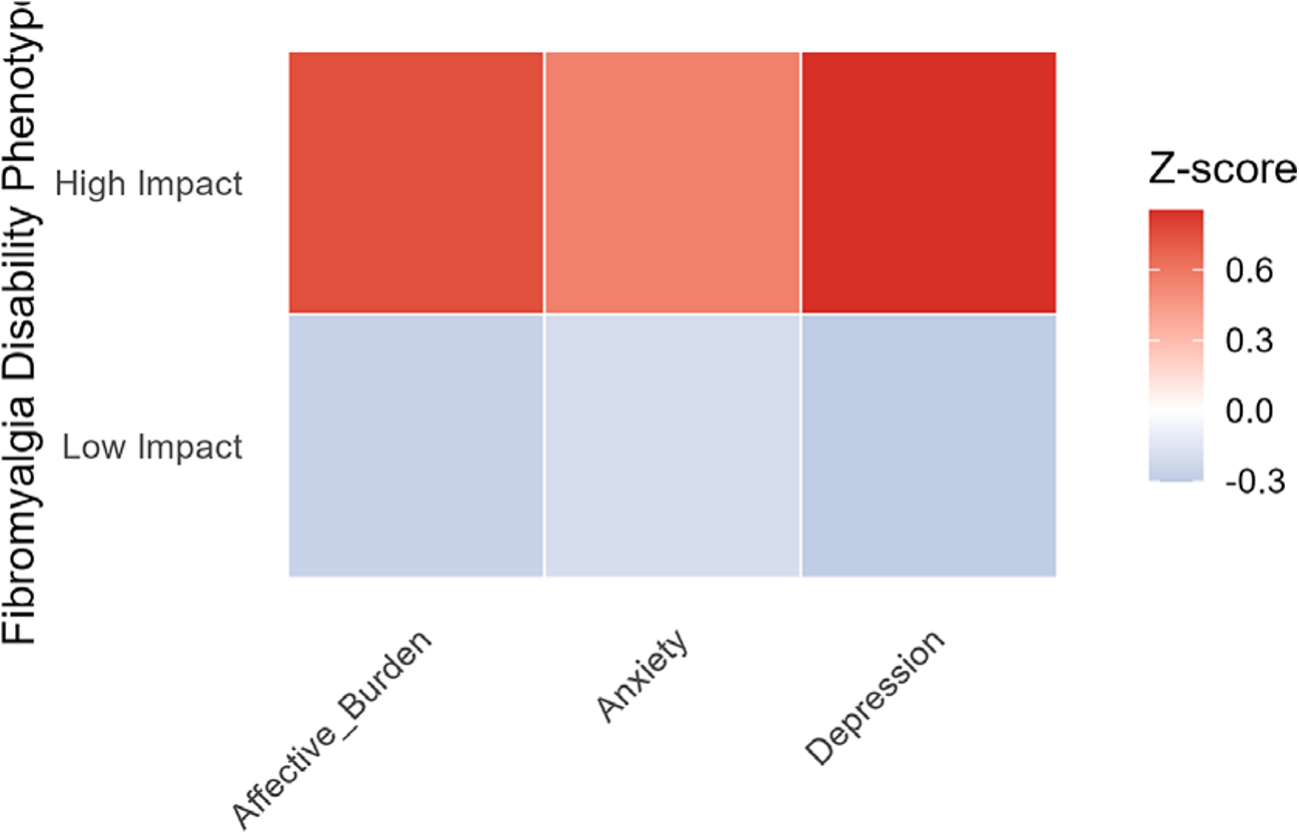
Psychiatric symptom domains across disability-based fibromyalgia phenotypes. *Note*: Heatmap illustrating standardized depressive symptoms, anxiety-related distress, and global affective burden across low-impact and high-impact fibromyalgia phenotypes. Values represent mean standardized z-scores for each symptom domain, with warmer colors indicating greater symptom burden.

**Figure 2.**
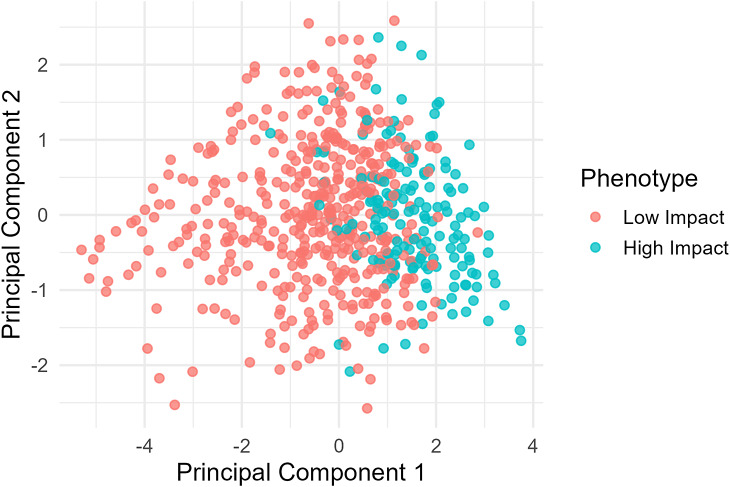
Partial orthogonality between affective burden and functional disability. *Note*: Principal component analysis of affective burden, functional disability, pain severity, and central sensitization demonstrates partial separation between affective and disability-related dimensions. Points are colored by disability-based fibromyalgia phenotype, supporting a multidimensional neuropsychiatric model of fibromyalgia. The first principal component explained 48.3% of total variance and loaded primarily on disability and pain variables, while the second principal component explained 27.1% of variance and loaded primarily on affective variables.

A heatmap illustrating psychiatric symptom domains across disability phenotypes is shown in Figure 1. Principal component analysis demonstrated partial separation between affective burden and functional disability, supporting partial orthogonality between these dimensions (Figure 2). The first principal component explained 48.3% of total variance and loaded primarily on disability and pain variables, while the second principal component explained 27.1% of variance and loaded primarily on affective variables.

## Discussion

This study demonstrates that psychiatric comorbidity in FM forms reproducible affective profiles that are not fully determined by disability severity. While higher affective burden was more common among individuals with greater functional impairment, substantial heterogeneity was observed within each disability stratum.

The identification of graded affective profiles supports a dimensional rather than categorical conceptualization of psychiatric comorbidity in FM. Importantly, the presence of minimal and mild affective symptom profiles among individuals with high-impact FM challenges unidimensional severity models and supports a neuropsychiatric framework in which affective dysregulation represents a partially independent axis of disease expression.^26^ ^,^^27^ ^,^^28^

These findings have direct clinical implications. Psychiatric symptom burden in FM should not be inferred solely from pain severity or functional impairment, as substantial affective heterogeneity exists within both low-impact and high-impact phenotypes. Systematic assessment of affective symptoms may improve diagnostic precision, risk stratification, and identification of patients who may benefit from targeted psychiatric or behavioral interventions, even in the absence of severe physical disability.

From a therapeutic perspective, affective profiling may inform more individualized treatment strategies. Patients with prominent affective symptom burden may benefit from integrated care models incorporating cognitive-behavioral therapy, mindfulness-based interventions, or pharmacologic treatments targeting mood and anxiety symptoms, whereas patients with minimal affective burden may respond adequately to pain-focused or functional rehabilitation approaches. These findings support a stratified, multidimensional treatment framework rather than a one-size-fits-all model for FM management.

Although disability was dichotomized using validated percentile-based thresholds to enhance clinical interpretability, affective symptom burden varied continuously across disability strata, reinforcing the conceptual distinction between functional impairment and psychiatric symptom expression. Complementary analyses treating disability as a continuous variable yielded consistent results, supporting robustness of the primary findings.

Conceptually, these results align with a multidimensional neuropsychiatric framework in which pain, disability, and affective symptoms represent partially overlapping but non-redundant domains contributing to FM heterogeneity.

### Strengths and limitations

Because certain Beck Depression Inventory items overlap with somatic symptoms common in FM (e.g., fatigue and sleep disturbance), we acknowledge the potential influence of symptom overlap on affective burden scores. However, inclusion of the SF-36 Emotional Well-Being subscale mitigates this concern.

Strengths include a large multisite cohort, harmonized measures, and a data-driven analytic approach. Limitations include reliance on self-report psychiatric measures and the cross-sectional design. Longitudinal studies integrating neurobiological markers will be important for future validation.

## Conclusions

Psychiatric comorbidity in FM reflects partially independent affective dimensions rather than a simple extension of disability severity. Recognizing affective profiles may enhance neuropsychiatric understanding, clinical stratification, and personalized intervention strategies.
